# Paramedic Education to Support the Use of Low-Acuity Care Pathways: A Scoping Review Protocol

**DOI:** 10.3390/nursrep13010025

**Published:** 2023-02-13

**Authors:** Anthony Carnicelli, Dale G. Edwards, Anne-Marie Williams

**Affiliations:** 1 Tasmanian School of Medicine, College of Health and Medicine, University of Tasmania, Hobart, TAS 7000, Australia; 2 School of Paramedicine, College of Health and Medicine, University of Tasmania, Hobart, TAS 7000, Australia

**Keywords:** paramedic, education, training, low acuity, alternative pathways

## Abstract

Ambulance services worldwide have transformed over time into health care services that not only attend to life-threatening emergencies, but are also increasingly being utilised for patients with low-acuity or non-urgent illness and injury. As a result, there has been a need to adapt and include mechanisms to assist paramedics in the assessment and management of such patients, including alternative pathways of care. However, it has been identified that education and training for paramedics in the care of low-acuity patients is limited. This study aims to identify potential gaps in the literature and inform further research, paramedic education and training, patient care guidelines, and policy. A scoping review will be conducted utilising the Joanna Briggs Institutes methodology. A range of relevant electronic databases will be searched along with the grey literature, using search terms related to paramedic education for low-acuity patient care pathways. The search results will be screened by two authors and presented in the PRISMA-ScR format, with articles presented in tabular format and analysed thematically. The results of this scoping review will inform further research exploring paramedic education, clinical guidelines, policy and experiences in the management of low-acuity patients.

## 1. Introduction

Traditionally, the role of an ambulance service has been to respond to calls for emergency care and transport patients to a hospital emergency department (ED) for definitive management [1,2]. However, over the past two to three decades this has changed, with many ambulance services around the world now not only attending to life-threatening and emergency conditions, but also very commonly, to patients calling for illnesses and injuries that are considered to be non-urgent or “low acuity” in nature [2,3,4]. Low-acuity conditions include (but are not limited to) soft tissue injuries; minor falls; musculoskeletal back pain; gastroenteritis symptoms; headaches, e.g., migraine; and dizziness or vertigo from benign causes [5]. In fact, the utilisation by the public of ambulance services for these low-acuity conditions, has been increasing both within Australia and internationally [1,3,4,5,6,7]. For example, it was reported by one state ambulance service in Australia that 53% of patients assessed by emergency paramedics were either non-acute (i.e., low acuity) or did not require transport to an ED [8]. In New Zealand (NZ), approximately 85% of patients attended to by ambulances were categorised as low acuity [3], and in a study from the United States of America (USA), 75% of patients transported to the ED were deemed to be low acuity [9]. 

As a consequence of the growing proportion of low acuity conditions attended to by ambulance services, there is a varying percentage of patients that are deemed to not require transport to hospital [3,6,10,11,12,13,14]. Whilst many of these patients can safely remain in the community, there is a cohort who still require some form of health or medical care [15]. However, due to difficulties accessing alternative options including primary care, they are often transported to the ED as a result [16]. Various estimates have been made regarding such medically unnecessary ambulance transports to the ED, including reports of anywhere between 11 to 61%, with many patients subsequently discharged without significant treatment or referral [7,17,18,19,20]. There is also a group of patients who, despite being transported, leave without being assessed [21]. Previous research has reported that almost 40% of patients transported by ambulance are triaged to non-urgent categories [22]. It has been suggested that transporting low-acuity patients by ambulance to the ED, contributes to overcrowding and delays access to more appropriate and/or timely care, such as that provided by a primary care physician [3,5,23]. Further, the rising demand for paramedics to attend to lower acuity calls is also recognised as a contributor to increased ambulance response times to more urgent calls [24]. 

Several initiatives have been implemented to reduce the burden of low-acuity calls requiring emergency ambulance attendance. This includes the integration of secondary triage into call taking and dispatching systems to determine if, at the time of the call, patients can be diverted to an alternative health care provider or use other means of transport to the ED if required [5,25]. Additionally, specialist extended care or community paramedic roles have emerged over recent years, which aim to reduce transport to hospital by utilising an expanded scope of practice to manage a range of low-acuity medical conditions in the community and/or refer them to other health care services [24].

To further assist non-specialist or general paramedics with the increasing need to manage low-acuity conditions in the field, often until the patient can access their primary care practitioner, ambulance services around the world have implemented several strategies for providing alternative care pathways. These pathways include various options to guide low-acuity treatment and referral (T&R), including on-scene triage and assessment tools, flowcharts, guidelines, protocols, and policy [26,27,28,29]. The range of low-acuity conditions they address and provide guidance on varies. Some focus on only one or a limited number of issues, whilst others have developed protocols for a multitude of conditions [26,28,29,30,31,32,33]. 

Several challenges have been identified with the implementation and use of T&R pathways into general paramedic practice, with deficiencies in training notably highlighted [24,28,29,34,35]. It has been reported that inadequate training is a barrier to their use in the field, with paramedics citing a lack of confidence and thus hesitancy to use them even for appropriately identified patients [15,28,36]. Further, paramedics feel that it could be dangerous for either themselves and/or the patient to implement T&R options, as they require significantly greater levels of judgement and decision making compared to just transporting all patients to hospital [15,24,28,36]. Concerns around the need for training in non-conveyance have been identified by several studies, and it has been suggested that training not only improves guideline compliance, but also confidence in decision making [24,29,33,37,38].

Whilst attending to low-acuity patients is often viewed as non-traditional paramedic work [39], it is acknowledged that part of the role is to provide non-urgent care and facilitate access to other health services [36]. However, it appears that education and training to support paramedic use of alternative care pathways is limited and requires investigation. The objective of this scoping review is to explore and conceptually map the existing literature related to the education and training provided to emergency ambulance paramedics in the use of alternative care pathways, such as guidelines, protocols, or other methods. Any identified gaps in the literature will inform further research, paramedic education and training, policy, as well as clinical practice including patient management guidelines. This review is the first stage of a larger research project investigating the experiences of paramedics managing patients presenting with low-acuity conditions, including the influences on their decision making and how these are supported through education, training, policy, and guidelines. 

## 2. Review Question

The review question is “what education and training is provided to paramedics to support their use of alternative care pathways when managing low acuity patients in the community?”. 

## 3. Materials and Methods

This scoping review will utilise the Joanna Briggs Institute’s (JBI) methodology for scoping reviews [40,41], as well as the framework proposed by Arksey and O’Malley and further adapted by Levac et al. [42,43]. A search of Open Science Framework (OSF), PROSPERO, JBI Evidence Synthesis, MEDLINE (PubMed), and the Cochrane Database of Systematic Reviews (CDSM), revealed that there are currently no published, in progress, or registered scoping or systematic reviews about this subject. 

### 3.1. Inclusion Criteria

#### 3.1.1. Participants

This review will consider articles identified as for or about paramedics working in ambulance services based on the Anglo-American emergency medical service (EMS) operational model [44,45]. To ensure an international perspective, the definition of paramedic is informed by Olaussen et al. [46] and defined as a non-physician out-of-hospital health care professional. Nurses who work in an ambulance service with a similar clinical role to a paramedic will also be included. For example, in some Scandinavian countries such as Sweden, registered nurses work alongside paramedics or EMTs and are responsible for the assessment and overall care of the patient [47,48,49,50].

Health care professionals working in an ambulance service but not considered paramedics or equivalent, or who do not provide a primary road-based ambulance response, will be excluded. This includes ambulance physicians, aeromedical retrieval flight paramedics, nurses, ambulance communications call-takers, ambulance communications dispatchers, and secondary triage clinicians.

#### 3.1.2. Concept

The concept of interest for this review is the education and training provided to paramedics in the use of low-acuity care pathways. Often termed “treat and refer” or alternative care pathways, they enable paramedics to provide on scene treatment and/or referral to other health care services, to avoid medically unnecessary transport where appropriate [15,26,27,31,51,52].

Results will be included if they examine or review the initial and/or ongoing education and training related to the use of low-acuity pathways, as well as the method of educational delivery, e.g., face-to-face, online, or blended learning. Results that make no mention or reference to the theme will be excluded. References to paramedic/EMT systems operating within the armed forces will also be excluded.

#### 3.1.3. Context

The context of this review is paramedics practicing in ambulance services based on the Anglo-American EMS model. This EMS model is present in several countries including, but not limited to, Australia, NZ, the United Kingdom (UK), USA, Canada, the Republic of Ireland, South Africa, and throughout the Middle East [53]. Some countries within Europe and Scandinavia have also adopted a similar EMS model, including the Netherlands and Sweden [54,55,56].

Articles reporting on Franco-German EMS-based models will be excluded. This model is present across many parts of Europe and utilises doctors, including emergency physicians and anaesthetists, to provide out-of-hospital care [44,45,53,57,58,59].

To differentiate between literature reporting on the two different EMS models, the titles, abstracts and, where necessary, the full text of the articles will be manually interrogated.

### 3.2. Type of Sources

This review will consider primary research studies utilising quantitative and qualitative study designs. Grey literature including government, industry, and professional reports, as well as research theses will also be included. These will be limited to the English language. Literature published between 2002 and 2022 will be included. This timeframe was chosen as from around 2002, the literature on out-of-hospital alternative care pathways was expanding [51,60,61,62,63,64].

### 3.3. Search Strategy

The search strategy will aim to locate both published and unpublished studies. To ensure the review captures a broad range of ambulance-related literature, including internationally, the following databases will be included: MEDLINE (PubMed), Scopus, Embase (Ovid), Emcare (Ovid), and CINAHL (EBSCO). A search for grey literature will include Google Scholar, ProQuest Dissertations, and Theses Global. The search strategy will be adapted for each included information source. Table 1 reflects an example applied to the Scopus database.

All search results will be collated and uploaded into the Covidence™ systematic review software (Veritas Health Innovation, Melbourne, Australia. Available at www.covidence.org (accessed on 27 October 2022) [65] and Endnote™ 20 (Clarivate™, Philadelphia, PA, USA) [66] with duplicates removed. Following a pilot test, titles and abstracts will then be screened by two reviewers (A.C. and D.G.E.) against the inclusion criteria. The full text of selected citations will then be assessed in detail for final inclusion in the review. The results of the search and the study inclusion process will be reported in accordance with the Preferred Reporting Items for Systematic Reviews and Meta-analyses extension for scoping reviews (PRISMA-ScR) [67].

### 3.4. Data Extraction

Data will be extracted using a table developed by the research team, an example of which can be seen in Table 2. The table will be piloted and modified as necessary during the review process to ensure all relevant results are extracted [42]. Data extraction will be conducted by one reviewer (A.C.) and verified by a second reviewer (D.G.E.) [42]. Any disagreements will be resolved through discussion, or with a third reviewer.

### 3.5. Data Analysis and Presentation

The results will be presented as follows. First, the Preferred Reporting Items for Systematic Reviews and Meta-Analysis (PRISMA) flow diagram will outline the quantity of retrieved and included articles and reports. Second, the included studies will be summarised and presented in tabular format. Third, a narrative summary will describe the findings, key themes, and concepts of the literature in relation to the study question and objective; as well as identify gaps in the research [68].

## 4. Discussion

A scoping review method was selected for this study, as the aim is to identify the volume of available literature in relation to the research question, map key concepts, and provide a broad descriptive overview of the subject, including an exploration of knowledge gaps [69].

The findings from this scoping review may identify potential gaps or areas where improvements to education and training may be required to further assist paramedics in the assessment, management, and referral of patients presenting with low-acuity medical conditions. It may also inform practice guidelines and non-conveyance policies to continue to support paramedic clinical decision making.

This review will only consider literature published in English, which may be a potential limitation, as studies on low-acuity care pathways for paramedics published in other languages will not be included. A further limitation may be the timeframe chosen, as any research published prior to 2002 will be omitted.

## 5. Conclusions

The findings from this scoping review will inform the direction of further research focusing on exploring paramedic decision making and experiences with the management of low-acuity patients, including a document analysis of clinical practice guidelines and semi-structured qualitative interviews.

## Figures and Tables

**Table 1:** Search strategy for Scopus. Search conducted on 16 November 2022.

Search	Query	Records Retrieved
#1	“paramedic” OR “ambulance officer” OR “ambulance staff” OR “emergency medical technician” OR “emt” OR “ambulance nurse”	149,623
#2	“education” OR “training” OR “professional development”	8,981,200
#3	“ambulance” OR “prehospital” OR “pre-hospital” OR “pre hospital” OR “out of hospital” OR “out-of-hospital” OR “emergency medical services” OR “ems”	352,669
#4	“low acuity” OR “non-urgent”	6437
#5	#1 AND #2 AND #3 AND #4	161
Limited to	English Language, published between 2002 and 2022	156

**Table 2:** Data Extraction Table.

**Authors/** **Year/Country**	**Topic/Focus/** **Purpose**	**Design/** **Sampling Method/** **Data Analysis Method**	**Context/Setting/** **Sample**	**Key Findings**	**Limitations/** **Gaps** **(Future Research)**

## Data Availability

Not applicable.
